# Formation of lipid bodies and changes in fatty acid composition upon pre-akinete formation in Arctic and Antarctic *Zygnema* (Zygnematophyceae, Streptophyta) strains

**DOI:** 10.1093/femsec/fiw096

**Published:** 2016-05-10

**Authors:** Martina Pichrtová, Erwann Arc, Wolfgang Stöggl, Ilse Kranner, Tomáš Hájek, Hubert Hackl, Andreas Holzinger

**Affiliations:** 1 Faculty of Science, Department of Botany, Charles University in Prague, Benátská 2, 128 01 Prague, Czech Republic; 2 Institute of Botany, Functional Plant Biology, University of Innsbruck, Sternwartestraße 15, 6020 Innsbruck, Austria; 3 Faculty of Science, University of South Bohemia, Branišovská 1760, 370 05 České Budějovice, Czech Republic; 4 Biocenter, Division of Bioinformatics, Medical University of Innsbruck, Innrain 80, 6020 Innsbruck, Austria

**Keywords:** desiccation stress, fatty acid methyl ester, lipids, nitrogen starvation, polar green microalgae

## Abstract

Filamentous green algae of the genus *Zygnema* (Zygnematophyceae, Streptophyta) are key components of polar hydro-terrestrial mats where they face various stressors including UV irradiation, freezing, desiccation and osmotic stress. Their vegetative cells can develop into pre-akinetes, i.e. reserve-rich, mature cells. We investigated lipid accumulation and fatty acid (FA) composition upon pre-akinete formation in an Arctic and an Antarctic *Zygnema* strain using transmission electron microscopy and gas chromatography coupled with mass spectrometry. Pre-akinetes formed after 9 weeks of cultivation in nitrogen-free medium, which was accompanied by massive accumulation of lipid bodies. The composition of FAs was similar in both strains, and α-linolenic acid (C18:3) dominated in young vegetative cells. Pre-akinete formation coincided with a significant change in FA composition. Oleic (C18:1) and linoleic (C18:2) acid increased the most (up to 17- and 8-fold, respectively). Small amounts of long-chain polyunsaturated FAs were also detected, e.g. arachidonic (C20:4) and eicosapentaenoic (C20:5) acid. Pre-akinetes exposed to desiccation at 86% relative humidity were able to recover maximum quantum yield of photosystem II, but desiccation had no major effect on FA composition. The results are discussed with regard to the capability of *Zygnema* spp. to thrive in extreme conditions.

## Abstract (precis)

Green algae *Zygnema* spp. survive in the Arctic and Antarctica as pre-akinetes, which are modified vegetative cells that accumulate lipids with oleic and linoleic acid being the main fatty acids.

## INTRODUCTION

Algae and cyanobacteria can be found in almost all polar terrestrial habitats, including wetlands, snow, ice, soil or rocks (Sheath *et al*. 1996; Kim, Klochkova and Kang 2008; Spijkerman *et al*. 2012; Skácelová *et al*. 2013). They represent important primary producers and possess various tolerance mechanisms that enable them to survive the multiple stresses that characterize these environments, such as freezing, freeze–thaw cycles, UV and high irradiation, and desiccation (Elster 2002). Most species of polar freshwater and terrestrial algae do not form spores, but are capable of surviving in the vegetative state (Sheath *et al*. 1996). Such stress-tolerant vegetative cells differ morphologically and physiologically from unstressed or cultured cells by accumulation of reserves such as lipid bodies, and by forming thick cell walls (Sheath *et al*. 1996; Kim, Klochkova and Kang 2008).

Modifications of vegetative cells were recently investigated in polar filamentous conjugating green algae of the genus *Zygnema* (Pichrtová, Hájek and Elster 2014, 2016; Pichrtová, Kulichová and Holzinger 2014). *Zygnema* spp. are very common to both the Arctic and Antarctica, where they form large mats in shallow meltwater streams and pools (Kim, Klochkova and Kang 2008; Holzinger, Roleda and Lütz 2009; Skácelová *et al*. 2013). Young vegetative cells of *Zygnema* spp. have two stellar chloroplasts and are highly vacuolated. When resources are limiting, they stop growing and gradually develop into ‘pre-akinetes’, which have been defined as modified vegetative cells with thick cell walls and mucilaginous pectic layers, reduced chloroplast lobes, lower physiological activity and accumulated storage compounds (McLean and Pessoney 1971; Fuller 2013; Herburger, Lewis and Holzinger 2015). Of the latter, lipid bodies are the most abundant and can be readily visualized by light and transmission electron microscopy (McLean and Pessoney 1970; Holzinger, Roleda and Lütz 2009; Fuller 2013; Kaplan *et al*. 2013; Pichrtová, Kulichová and Holzinger 2014). Importantly, well-developed pre-akinetes are more stress tolerant than young vegetative cells. When hardened, pre-akinetes can tolerate osmotic stress (Kaplan *et al*. 2013; Pichrtová, Hájek and Elster 2014), slow desiccation (Pichrtová, Kulichová and Holzinger 2014) and they also serve as overwintering stages (Pichrtová, Hájek and Elster 2016). This is in agreement with reports on other algae, in which mature, starved cells were found to be more stress tolerant than young cells from nutrient-sufficient log-phase cultures (e.g. Nagao *et al*. 1999). Cells with lipid bodies and thick cell walls were also described from various natural stress environments (Morison and Sheath 1985; Darling, Friedmann and Broady 1987; Hoppert *et al*. 2004). Interestingly, accumulation of lipids was also observed in desiccation-tolerant, thick-walled cells in bryophyte protonemata (Rowntree *et al*. 2007).

Under laboratory conditions, the formation of lipid-rich cells can be easily induced by nitrogen deprivation (Goncalves, Johnson and Rathinasabapathi 2013; Abe *et al*. 2014; Pichrtová, Kulichová and Holzinger 2014; Ruiz-Domínguez *et al*. 2015; Zhu *et al*. 2015). Other stress factors also induce lipid accumulation and influence lipid and fatty acid (FA) content and composition (recently reviewed by e.g. Guschina and Harwood 2006, Vítová *et al*. 2014). Microalgae from cold environments, including polar and alpine species, have evolved to maintain membrane fluidity at low temperatures, and low temperature stress increases the abundance of polyunsaturated fatty acids (PUFAs) and short-chain or branched FAs (Morgan-Kiss *et al*. 2006). Desiccation stress has also been shown to promote accumulation of triacylglycerols (TAGs) in *Chlorella kesslerii* (Shiratake *et al*. 2013). Exposure to stress factors, most commonly nitrogen deprivation, is generally applied in algal biotechnology to induce lipid accumulation (Sharma, Schuhmann and Schenk 2012), and there is increasing commercial interest in using algae as promising sources of biofuel or high-value PUFAs (reviewed e.g. by Guschina and Harwood 2006, Sharma, Schuhmann and Schenk 2012). However, our knowledge of FA composition of conjugating green algae is only very limited (Lang *et al*. 2011). Our major aim was to conduct a detailed analysis of the FA composition of an Arctic and an Antarctic *Zygnema* spp. in which pre-akinete formation and lipid accumulation were observed (Pichrtová, Kulichová and Holzinger 2014; Pichrtová, Hájek and Elster 2016). We tested if FA composition changes upon the formation of pre-akinetes and desiccation stress. The results are discussed in the context of survival and development under the harsh environmental conditions of the Arctic and Antarctica.

## MATERIALS AND METHODS

### Algal material and cultivation

The Arctic *Zygnema* sp. strain ‘B’ (CCALA 976; Pichrtova 2011/1) and the Antarctic *Zygnema* sp. ‘C’ (CCALA 880; Snokhousova et Elster 2009/8) described in Pichrtová, Kulichová and Holzinger (2014) were used for the experiments. For detailed information including description of collection sites and phylogenetic position of the strains, see Pichrtová, Kulichová and Holzinger (2014). Cultures were maintained on Bold's Basal Medium (BBM; Bischoff and Bold 1963) solidified with 1.5% agar and incubated under optimal growth conditions, 18°C and continuous light (35 μmol m^−2^ s^−1^). Hereafter, these cultures are referred to as ‘young cultures’.

### Induction of pre-akinetes, desiccation and harvest of algal cultures

To induce the formation of pre-akinetes and lipid accumulation, the cultures were transferred to agar plates with BBM without nitrate or any other source of nitrogen, and kept at 18°C and continuous light (35 μmol m^−2^ s^−1^) for 9 weeks as previously described (Pichrtová, Kulichová and Holzinger 2014). Pre-akinete cultures are referred to as ‘mature cultures’.

After 9 weeks of nitrogen depletion, thin layers of pre-akinete filaments were spread on glass-fiber filters (GE Healthcare, Little Chalfont, UK; 4.7 cm in diameter) and placed into desiccation chambers either over saturated KCl (86% relative air humidity, RH) or partly dried silica gel (18% RH). Each chamber was equipped with a small electric fan allowing fast (<12 h) water content equilibration of the samples. After 24 h of desiccation, the algal material was rehydrated by adding 2 mL of water, and transferred into another chamber above distilled water (98%–100% RH) for another 24 h, and the proportion of surviving cells was estimated using a light microscope. All experiments were performed at room temperature.

For further analysis, the biomass of young, nutrient-sufficient 2-week-old cultures and of mature cultures were harvested. The latter were also investigated at five different stages of the desiccation/recovery experiment: (i) prior to desiccation treatment, (ii) after desiccation above saturated KCl or (iii) silica gel, and after rehydration following the desiccation over (iv) saturated KCl solution and (v) silica gel. The filters with biomass were placed into 2 mL micro tubes (Eppendorf, Hamburg, Germany), immediately frozen in liquid nitrogen, freeze-dried and stored at −80°C prior to analyses. For each combination of strain and treatment, five independent replicates were obtained.

### Chlorophyll *a* fluorescence

Chlorophyll *a* fluorescence was measured by an imaging-modulated fluorimeter FluorCam (PSI, Czech Republic). The minimum fluorescence of dark-acclimated cultures (*F*_0_) and the maximum fluorescence after the application of a saturation pulse (*F*_M_) were recorded from each sample spread on a filter. Maximum quantum yield of photosystem II photochemistry (*F*_V_/*F*_M_ = (*F*_M_ − *F*_0_) / *F*_M_) was computed in order to characterize the physiological state of the cultures (Maxwell and Johnson 2000). *F*_V_/*F*_M_ of the cultures was measured for all cultures before desiccation and after recovery (*n* = 5).

### Gas chromatography coupled with mass spectrometry

FAs were derivatized to FA methyl esters as described by Li-Beisson *et al*. (2010). Briefly, about 5 mg of freeze-dried sample were treated with 2 mL of methanol:toluene:sulfuric acid 10:3:0.25 (v:v:v) containing 0.01% (w:v) butylated hydroxytoluene. A total of 200 μg of heptadecanoic acid (C17:0, dissolved in hexane) was added simultaneously as internal standard. Samples were incubated at 80°C for 90 min with constant agitation before adding 760 μL of hexane and 2.3 mL of 0.9% NaCl (w:v). Samples were vigorously mixed before centrifugation for 10 min at 3000 × *g*. The supernatant was transferred to autosampler vials and kept at −20°C if not analyzed immediately. FA methyl esters were separated using a Trace 1300 gas chromatograph (Thermo-Scientific, Waltham, USA) on a 30 m BPX70 column (SGE Analytical Science #054622, Melbourne, Australia) and detected using a TSQ 8000 triple quadrupole mass spectrometer (Thermo-Scientific) operated in full scan mode (50–550 m z^−1^). The temperature gradient was first set to 110°C for 1 min, then increased by 9°C min^−1^ up to 180°C, and finally increased by 15°C min^−1^ up to 230°C, which was held for 5 min with a carrier gas flow of 1.5 mL min^−1^ of helium and a split ratio set to 50. The ion source temperature was set to 250°C and the transfer line to 240°C. A commercial FA methyl ester mix (Sigma Aldrich ref. 18919, Missouri, USA) was used to confirm the identities of the FAs. External standards of palmitic, stearic, oleic, linoleic and α-linolenic acid were used in conjunction with the internal standard to determine the total amount of each of these FAs. Data analysis was performed using the Xcalibur software (Thermo-Scientific).

### Transmission electron microscopy

For transmission electron microscopy, young *Zygnema* sp. of strains B and C were prepared according to Aichinger and Lütz-Meindl (2005) with modifications by Pichrtová *et al*. (2013). Briefly, samples were high-pressure frozen in 150 mM sucrose and freeze substitution was carried out in 2% OsO_4_ in acetone at −80°C; samples were then gradually transferred via propylene oxide to Agar Low Viscosity Resin (Agar Scientific R 1078, Essex, UK). Mature cells were chemically fixed according to Holzinger, Roleda and Lütz (2009). Samples were fixed for 1 h in 2.5% glutaraldehyde in 20 mM cacodylate buffer (pH = 6.8), rinsed and postfixed in 1% OsO_4_ at 4°C for 18 h. Samples were dehydrated in increasing ethanol concentrations, embedded in modified Spurr's resin (Ellis 2006). Ultrathin sections were counterstained with uranyl acetate and Reynold's lead citrate and viewed with a Zeiss Libra 120 (Zeiss, Oberkochen, Germany) transmission electron microscope at 80 kV. Digital images were captured with a TRS (Tröndle Restlicht Verstärker Systeme, Moorenweis, Germany) 2k SSCCD camera controlled by OSIS iTEM software and further processed with Adobe Photoshop Elements 11.

### Statistical analyses

*F*_V_/*F*_M_ data were analyzed using the general linear model factorial analysis of variance (ANOVA). The two factors ‘strain’ and ‘treatment’ were considered as factors with fixed effects. Additionally, Tukey's test was performed for *post hoc* pairwise comparisons.

Principal component analysis was used to visualize correlations between relative abundance of individual FAs and genotypes/treatments.

Differences in the absolute content of individual FAs were tested using two-way ANOVA (factors ‘genotype’ and ‘culture age’). In case a significant interaction between both factors was detected, differences between the young and mature cultures were compared separately for each genotype using Welch two-sample t-test with Bonferroni correction.

Changes in FA composition during desiccation/rehydration were tested by two-way ANOVA. In cases where no statistical interaction between genotype and treatment was observed, FA levels in both genotypes were normalized to the mean of the respective not desiccated group and then combined. To compare FA relative abundance between treatment groups and the non-desiccated condition, a one-way ANOVA followed by Dunnett's *post hoc* tests was performed.

The analyses were performed in Statistica 12 (Statsoft) and R 3.2.1 (including the packages ‘beeswarm’ and ‘multcomp’). All tests were two-sided and the assumption of normal distribution was tested.

## RESULTS

### Ultrastructure of young and mature cultures

**Figure 1.**
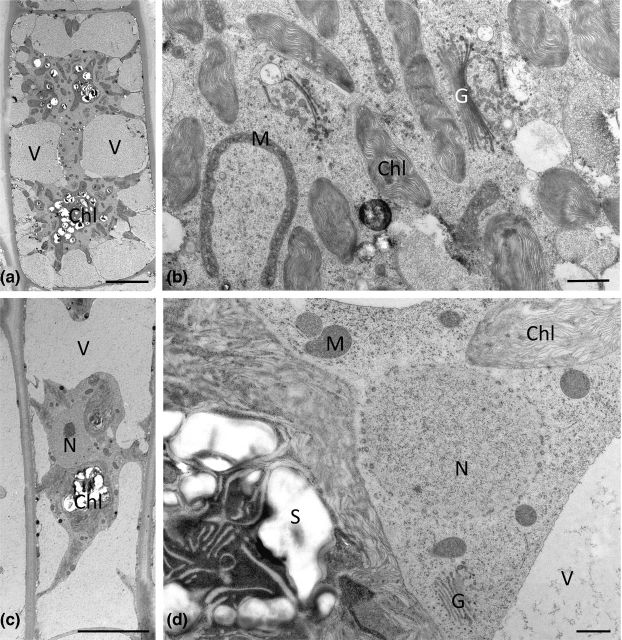
Transmission electron micrographs of young vegetative *Zygnema* sp. cells of strains B (**a**, **b**) and C (**c**, **d**). (a) Overview showing a high degree of vacuolization and two stellate chloroplasts, (b) cytoplasmic detail with numerous chloroplast lobes, Golgi bodies and mitochondrion, (c) overview with central nucleus and large vacuoles, (d) detail of the cell center with nucleus, Golgi body, mitochondria, chloroplast with starch grains surrounding the pyrenoid. Abbreviations: Chl chloroplast, G Golgi body, M mitochondrion, N nucleus, S starch, V vacuole. Bars: a, c 10 μm; b, d 1 μm.

Young vegetative cells of strain B showed a high degree of vacuolization (Fig. 1a). The two stellate chloroplasts contained pyrenoids with starch grains and were surrounded by a thin layer of cytoplasm (Fig. 1a). Within the cytoplasm, multiple sections through the chloroplast lobes with parallel-arranged thylakoid membranes were found (Fig. 1b), and numerous Golgi bodies and long mitochondria indicated that the cells were metabolically active (Fig. 1b). Cells of strain C also showed a large degree of vacuolization (Fig. 1c) and the pyrenoids were surrounded by massive starch grains (Fig. 1d). The cytoplasm contained a centrally arranged nucleus, surrounded by numerous mitochondria and Golgi bodies (Fig. 1d). The cytoplasm of both strains was dense, and virtually no lipid bodies were detected in young cultures (Fig. 1b and d).

**Figure 2.**
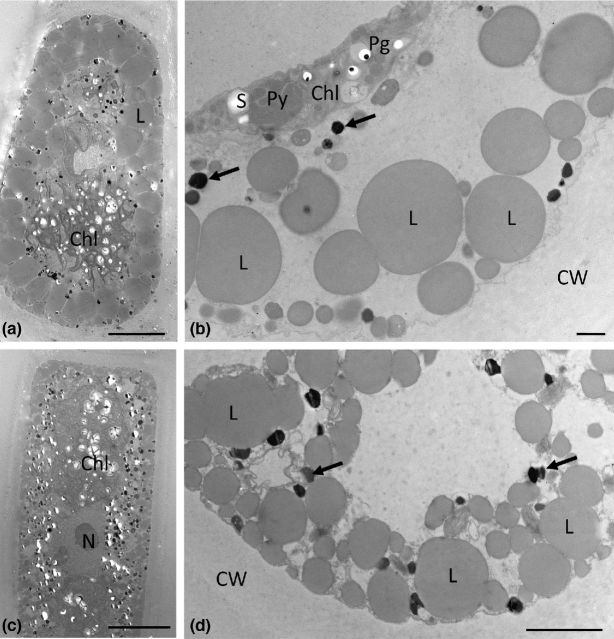
Transmission electron micrographs of mature vegetative cells of *Zygnema* sp. strains B (**a**, **b**) and C (**c**, **d**). (a) Overview showing the cell periphery filled with lipid bodies, and central chloroplasts; (b) detail of the cell periphery with cell wall, lipid bodies and electron-dense particles (arrows); the chloroplast contains starch grains and numerous plastoglobules: (c) overview with central nucleus and chloroplasts; lipid bodies and electron-dense particles in the cell periphery: (d) lipid bodies and electron-dense particles (arrows). Abbreviations: Chl chloroplast, CW cell wall, N nucleus, Pg plastoglobule, Py pyrenoid, S starch. Bars: a, c 10 μm; b, d 1 μm.

By contrast, lumina of mature cells of both strains were filled with large (up to 4 μm) lipid bodies with medium electron density and only little vacuolization remained (Fig. 2a–d). The chloroplasts were reduced, but the pyrenoids still contained starch grains and numerous plastoglobules were present (Fig. 2b). In the cytoplasm of both strains, many electron-dense particles were found in addition to the lipid bodies (Fig. 2b and d).

### Physiology of young and mature cells, and response to desiccation stress

**Figure 3.**
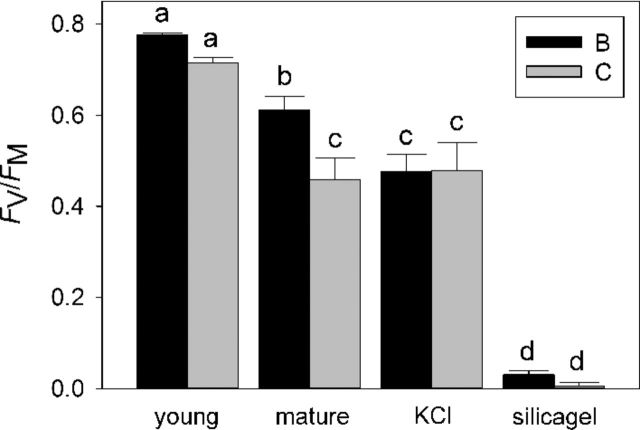
Maximum quantum yield of photosystem II (*F*_V_/*F*_M_) of young and mature cultures of *Zygnema* sp. strains B and C prior to desiccation, and of mature cultures that were rehydrated after desiccation above KCl (86% RH) or silicagel (18% RH); mean +SD, *n* = 5. Different letters represent statistical differences between means (*P* < 0.01; general linear model factorial ANOVA, Tukey's *post hoc* tests).

Young and mature cells also clearly differed in their physiological performance. Cultures starved for 9 weeks had significantly lower maximum quantum yield of photosystem II (*F*_V_/*F*_M_) than young ones, and this difference was more pronounced in strain C than in B (Fig. 3). Upon rehydration following desiccation at 18% RH, the low *F*_V_/*F*_M_ values (Fig. 3) indicated that neither of the strains was able to survive this treatment, and this was confirmed by light microscopy, which showed disintegrated or empty cells (Fig. S1b, Supporting Information). In contrast, both strains survived desiccation above saturated KCl. Mature cells of strain C fully recovered *F*_V_/*F*_M_, and those of strain B recovered 78% of their initial *F*_V_/*F*_M_ values (Fig. 3). In both strains, light microscopy showed that about 50% of the cells survived desiccation over KCl (Fig. S1a, Supporting Information).

### FA composition of young and mature cultures

**Figure 4.**
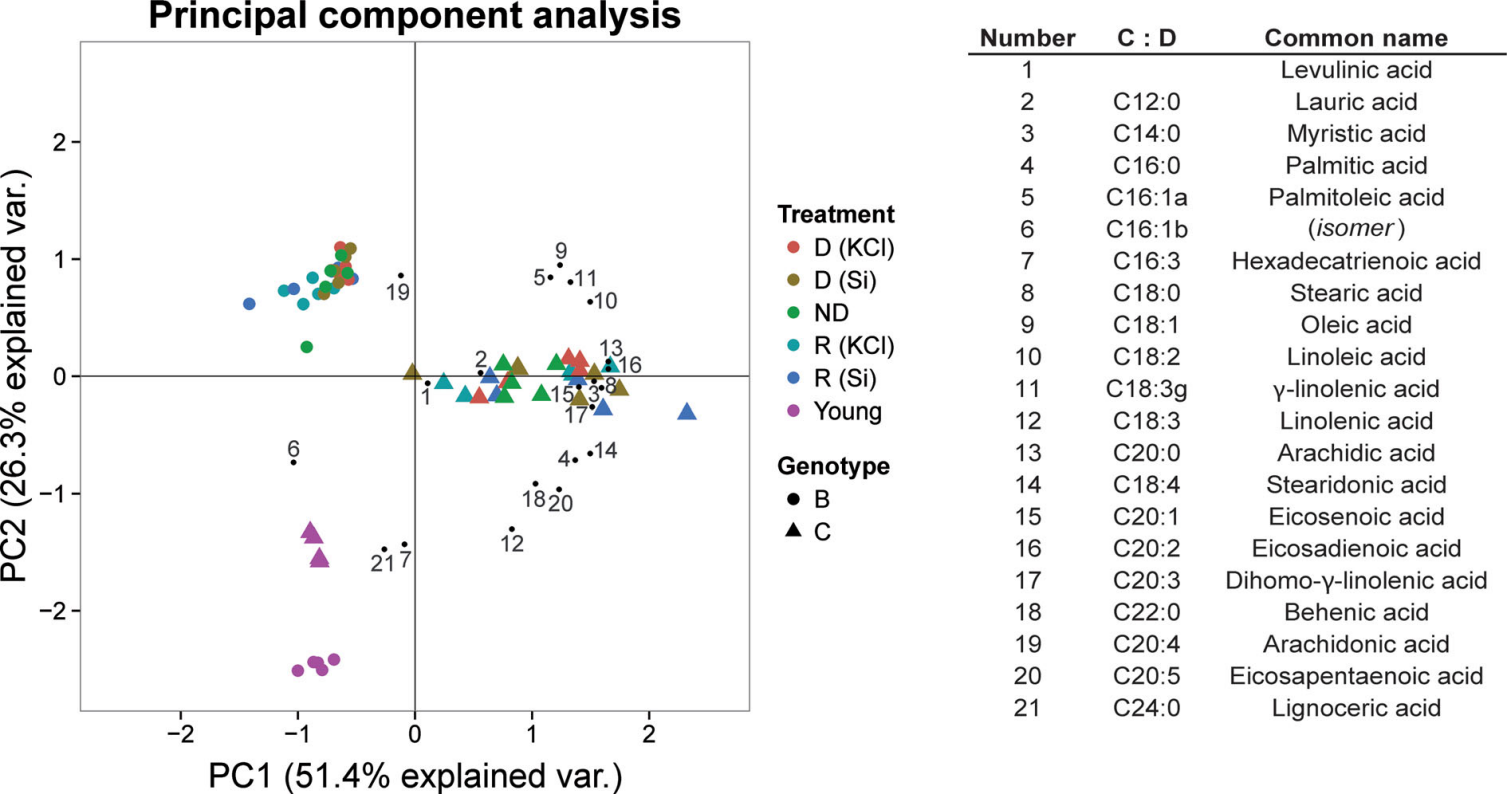
PCA ordination plot of samples from both strains and all experimental treatments based on their relative FA composition. Abbreviations: D (KCl): desiccated over KCl, D (Si): desiccated over silicagel, ND: not desiccated, R (KCl): desiccated over KCl and rehydrated, R (Si): desiccated over silicagel and rehydrated, Young: young cultures. The first two axes are shown, for additional two axes see Fig. S3, Supporting Information.

Principal component analysis based on the relative FA composition separated all samples into four clusters along the first two axes that together account for 78% of total variation (Fig. 4). The first principal component (PC1) separated pre-akinete cultures of *Zygnema* sp. C from all other samples. In addition, the second principal component (PC2) clearly separated all pre-akinete cultures from all young ones while simultaneously grouping the samples by strain. The young samples of both strains were characterized by high relative proportion of hexadecatrienoic (C16:3, no. 7) and lignoceric (C24:0, no. 21) acid. On the other hand, high relative proportions of oleic (C18:1, no. 9), linoleic (C18:2, no. 10) and γ-linolenic (C18:3g, no. 11) acid discriminated mature cells (Fig. 4).

**Figure 5.**
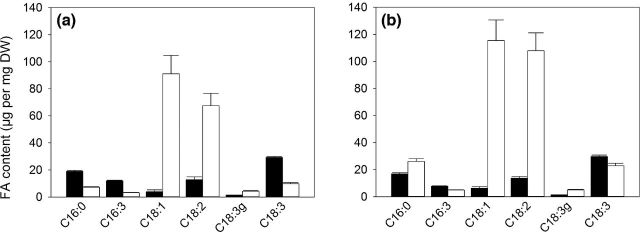
Absolute quantification of fatty acid content in *Zygnema* sp. B (**a**) and *Zygnema* sp. C (**b**). Black bars show young cultures and white bars mature cultures. The FAs shown here were quantified using external standards; relative quantification data is also available for all other detected compounds (Fig. S2, Supporting Information). Data are means plus standard deviation (*n* = 5). Welch's two sample t-test with Bonferroni correction proved that the content of each fatty acid (within strain) was significantly different between young and mature cultures (*P* < 0.001).

Absolute quantification of FA content revealed that young cultures of both species shared a very similar overall pattern in the content of dominant FAs quantified using external standards, even though their abundance slightly differed (Fig. 5). The dominant FAs in young cells of both strains were palmitic (C16:0), hexadecatrienoic (C16:3), linoleic (C18:2) and α-linolenic (C18:3) acid (Fig. 5). In mature cells of both strains, massive increases in contents of oleic (C18:1) and linoleic (C18:2) acid were detected (Fig. 5). In contrast, the content of α-linolenic (C18:3) acid, the most abundant compound in young cells, decreased during starvation.

Proportions of all detected FAs were estimated based on the respective peak areas (Fig. S2, Supporting Information). Palmitic (C16:0), hexadecatrienoic (C16:3), linoleic (C18:2) and α-linolenic (C18:3) acid accounted for more than 80% of detected FAs in young cells (Fig. S2a, Supporting Information). In pre-akinetes, a similar proportion was made up by the two dominant FAs, oleic (C18:1) and linoleic (C18:2) acid; Fig. S2b, Supporting Information. Other important FAs in young samples were oleic (C18:1), stearidonic (C18:4) and eicosapentaenoic (C20:5) acid (Fig. S2a, Supporting Information).

### Desiccation and rehydration treatment

**Figure 6.**
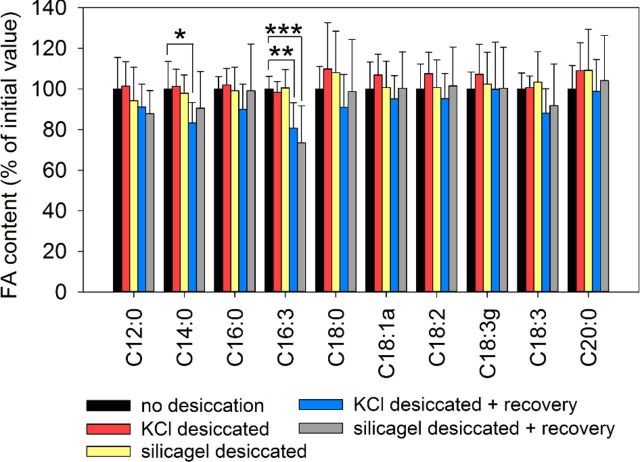
Fatty acid content in different experimental treatments relative to non-desiccated control. Only data for the most abundant fatty acids are shown. As two-way ANOVA did not reveal any significant interaction between strain and treatment, data for both strains were analyzed together (mean +SD, *n* = 10). Significant differences from the initial value (no desiccation) are marked by asterisks (*P* < 0.001***, 0.001 < *P* < 0.01**, 0.01 < *P* < 0.05*, Dunnett's test).

No major changes in the content of the most abundant FAs during desiccation or rehydration could be detected (Fig. 6). The content of some FAs was lower after desiccation and rehydration than in non-desiccated samples. However, this decline was statistically significant only in hexadecatrienoic (desiccated at 86% and 18% RH) and myristic acid (desiccated at 86% RH; Fig. 6). A similar conclusion could be drawn on comparing relative content of all FAs. All mature samples were clearly separated only by strain (B or C), but samples from different desiccation/rehydration treatments were randomly scattered within those clusters (Fig. 4). No clear separation of desiccated and non-desiccated samples was shown even along other PC axes, only the rehydrated samples clustered together (Fig. S3, Supporting Information).

## DISCUSSION

### Lipid bodies accumulate in pre-akinetes

As a result of nitrogen starvation, polar *Zygnema* sp. strains form pre-akinetes, which are filled with lipid bodies and after hardening, are capable of surviving desiccation (Pichrtová, Kulichová and Holzinger 2014). In this study, we characterized FA composition of young cells and pre-akinetes in response to starvation and desiccation stress.

In both strains, the Arctic one and the Antarctic one, a high degree of vacuolization of young cells, but hardly any lipid bodies or other storage compounds were observed, in agreement with previous work on other *Zygnema* spp. (McLean and Pessoney 1971; Herburger, Lewis and Holzinger 2015). When these cultures were transferred to nitrate-free agar medium, pre-akinetes with large lipid bodies were formed (Pichrtová, Kulichová and Holzinger 2014). Alpine *Zygnema* strains also accumulated lipids after several months of culture in medium in which nutrients became depleted over time (Herburger, Lewis and Holzinger 2015). Lipid bodies were also found in field samples, for example in *Zygnema* sp. from Svalbard at the end of vegetation season (Holzinger, Roleda and Lütz 2009; Pichrtová, Hájek and Elster 2016). In samples from India, it was shown that the oil contributed to 8.3% of the dry mass (Sawarkar and Nandkar 2013).

Accumulation of neutral lipids, predominantly TAGs, is characteristic of starved or otherwise stressed cells and hence, TAGs are often observed in algal cultures in the stationary phase (Goncalves, Johnson and Rathinasabapathi 2013; Abe *et al*. 2014). Nutrient-deficient algae continue synthesizing FAs which, if not needed as building blocks of new membranes, are used for the production of TAGs (Thompson 1996). The accumulation of N-free lipids (and carbohydrates) under stress conditions is thought to result from a shift in photosynthate allocation from growth to energy storage (Solovchenko 2012; Vítová *et al*. 2014). In addition, plastid membranes could be actively degraded and the released FAs incorporated into TAGs (Miller *et al*. 2010; Boyle *et al*. 2012). Recycling of polar membrane lipids into lipid bodies was also detected by radiolabeling in *C. protothecoides* (Goncalves, Johnson and Rathinasabapathi 2013). Upon pre-akinete formation, we observed chloroplast degradation in conjunction with the accumulation of plastoglobules in starved cells of both strains (Fig. 2). Accordingly, *F*_V_/*F*_M_ was significantly reduced in pre-akinetes, in agreement with previous studies, which showed that pre-akinete formation coincides with lowered effective quantum yield (Pichrtová, Kulichová and Holzinger 2014; Herburger, Lewis and Holzinger 2015).

### Oleic acid and linoleic acid increase most upon pre-akinete formation

FA distribution patterns reflect phylogenetic relationships and can be used as chemotaxonomic markers, and the FAs composition of the two *Zygnema* sp. strains is typical of green algae (Graeve *et al*. 2002; Lang *et al*. 2011). The predominant FAs in both strains were palmitic (C16:0), hexadecatrienoic (C16:3), oleic (C18:1), linoleic (C18:2) and α-linolenic (C18:3) acid (Fig. 5; Fig. S2, Supporting Information). An extensive screening of FAs composition across various algal groups showed that palmitic acid is a typical compound of cyanobacteria and primary plastid groups (Lang *et al*. 2011), within which the division Streptophyta is characterized by high proportions of oleic acid (Lang *et al*. 2011). The FA composition was very similar in both strains, albeit some differences in relative abundance of the less abundant FAs were found, separating the two strains in the PCA analysis (Fig. 4). Variation in individual FAs was also found in closely related species or isolates of the same species by Lang *et al*. (2011). For example, some *Closterium* strains (Zygnematophyceae) contained high amounts of γ-linolenic acid, whereas it was absent in others (Lang *et al*. 2011).

FAs are the building blocks of phospholipids, glycolipids and betaine lipids, which are integral parts of membranes (Zhu *et al*. 2015). Young cultures had virtually no lipid bodies, and palmitic, hexadecatrienoic and α-linolenic acid, typical compounds of membrane lipids (Kumari *et al*. 2013), were the dominant FAs (Fig. 5). Upon pre-akinete formation, typical membrane lipids such as hexadecatrienoic acid and α-linolenic acid became less abundant, as also found in other nutrient-deficient green algae (Arisz *et al*. 2000; Boyle *et al*. 2012), whereas FAs typical of storage lipids increased. The most striking increase was found for oleic (C18:1) and linoleic (C18:2) acid, which rose up to 17-fold and 8-fold, respectively. Accumulation of these two FAs was also found as a result of nitrogen depletion in aeroterrestrial *Coccomyxa* sp. (Abe *et al*. 2014), *Chlamydomonas moewusii* (Arisz *et al*. 2000) and *Ch. reinhardtii* (Boyle *et al*. 2012). Oleic acid, a typical component of neutral lipids (Thompson 1996; Arisz *et al*. 2000; Zhu *et al*. 2015) also accumulated in snow algae (Spijkerman *et al*. 2012) and *C. zofingiensis* (Zhu *et al*. 2015). Hence, the changes in FA composition reflect the transition in photosynthate allocation from growth processes to energy storage.

In general, increase in saturated and monounsaturated FAs and decrease in PUFA contents accompany nitrogen starvation (Abe *et al*. 2014). However, the absolute content of PUFAs in our samples did not change consistently with pre-akinete formation. Hexadecatrienoic and α-linolenic acid were more abundant in fresh cultures, and γ-linolenic and arachidonic acid in mature cultures. *Zygnema* spp. also contained stearidonic (C18:4), eicosatrienoic (C20:3), arachidonic (C20:4) and eicosapentaenoic (C20:5) acid. Long-chain PUFAs (C > 18) are interesting compounds for biotechnological applications commonly found mainly in red and chromalveolate algae (Graeve *et al*. 2002; Lang *et al*. 2011). Markedly, no traces of these compounds were found in the closely related streptophytic alga *Klebsormidium* from Antarctica (Teoh *et al*. 2004). Other cultivation conditions than nitrogen availability have also an important influence on FA unsaturation, typically low temperature (Morgan-Kiss *et al*. 2006). In our study, the algae were kept at 18°C and therefore it can be assumed that field-collected samples or cultures maintained at low cultivation temperature would have a slightly different FA composition.

Furthermore, we investigated if desiccation stress leads to additional changes in FA composition in pre-akinetes. Air drying was used to stimulate TAG production in biotechnologically interesting strains (Shiratake *et al*. 2013). For example, when exposed to 98% RH, *C. kessleri* showed increased FAs production. In contrast, we did not find elevated levels of FAs in either of the *Zygnem*a strains exposed to desiccation (Fig. 6). In agreement with Pichrtová, Kulichová and Holzinger (2014), pre-akinetes of both strains survived mild desiccation stress at 86% RH. Strain C was described as more desiccation tolerant than strain B in Pichrtová, Kulichová and Holzinger (2014) and here, fully recovered initial *F*_V_/*F*_M_ values after desiccation at 86% RH whereas strain B recovered 78% of initial *F*_V_/*F*_M_. However, desiccation at 18% RH was lethal. In both strains, neither desiccation nor rehydration caused substantial changes in FA contents (Fig. 6). Putative separation of rehydrated samples (Fig. S3, Supporting Information) is probably only due to the effect of FA degradation in dead cells, because samples desiccated at 86% RH cluster together with those desiccated at 18% RH.

### *Zygnema* spp. in polar habitats

Massive accumulation of storage compounds in response to nitrogen starvation is not a general feature of all algae, being dependent on the taxon-specific strategies of carbon allocation (Giordano, Palmucci and Norici 2015). However, some species including *Zygnema* spp. produce pre-akinetes, which are mature, vegetative cells that develop upon nutrient depletion, accumulate lipids and can be hardened to the stresses that accompany the end of their growing season. Apparently, this ability to accumulate storage compounds upon seasonal changes of environmental factors is advantageous in the unstable conditions of polar terrestrial environments. Pre-akinete formation allows year-to-year survival of vegetative cells, without production of specialized resistant cells during the short and cold polar summer (Pichrtová, Hájek and Elster 2016). Lipid accumulation serves as a source of energy and carbon and as a supply of FAs necessary for rearrangements of the membranes (Vítová *et al*. 2014). Therefore, lipid storage may provide the species with a competitive advantage during early polar summer (Davey 1988) as pre-akinetes may utilize their storage to supplement photosynthetic carbon assimilation and support rapid growth under nutrient abundance.

## Supplementary Material

Supplementary Data
