# Supplementary figures and images for: Formation of lipid bodies and changes in fatty acid composition upon pre-akinete formation in Arctic and Antarctic Zygnema (Zygnematophyceae, Streptophyta) strains
Source: FEMS Microbiol Ecol. 2016 May 10;92(7):fiw096. doi: 10.1093/femsec/fiw096 (PMC4892695; doi:10.1093/femsec/fiw096)

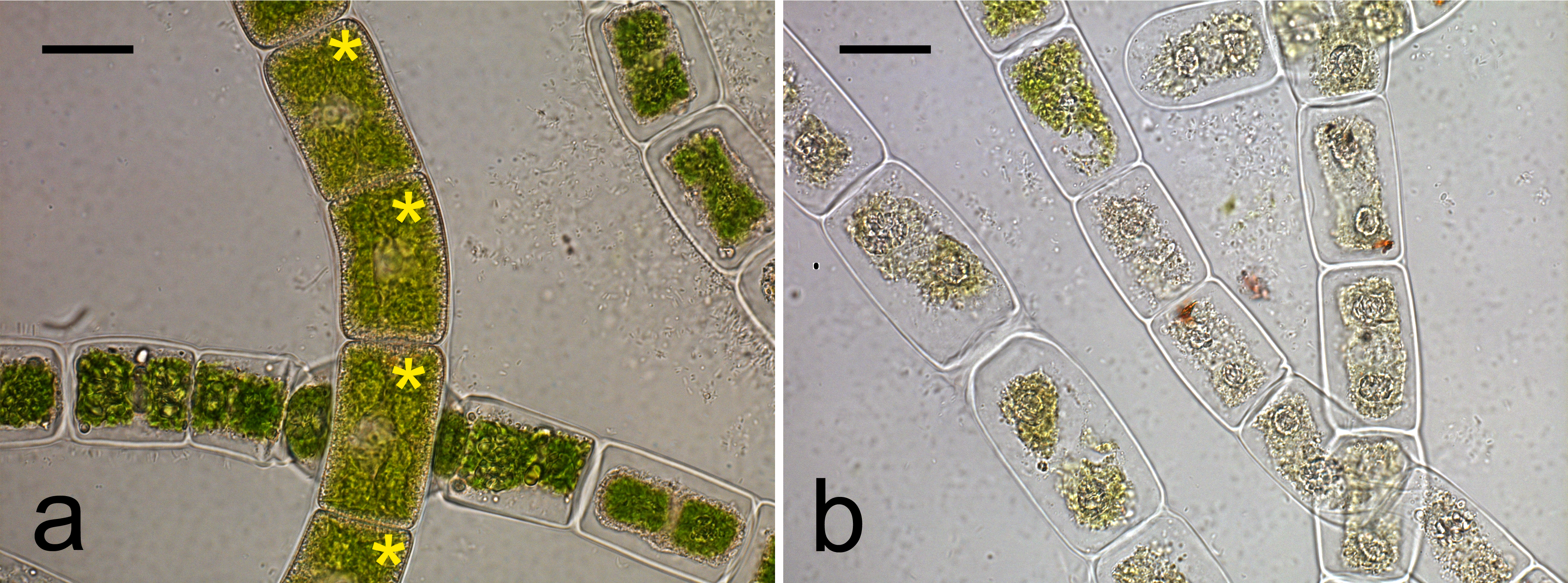

Supplement: Supplementary Data [file fiw096_supplementary_data.zip › Fig_supl_1.tif]

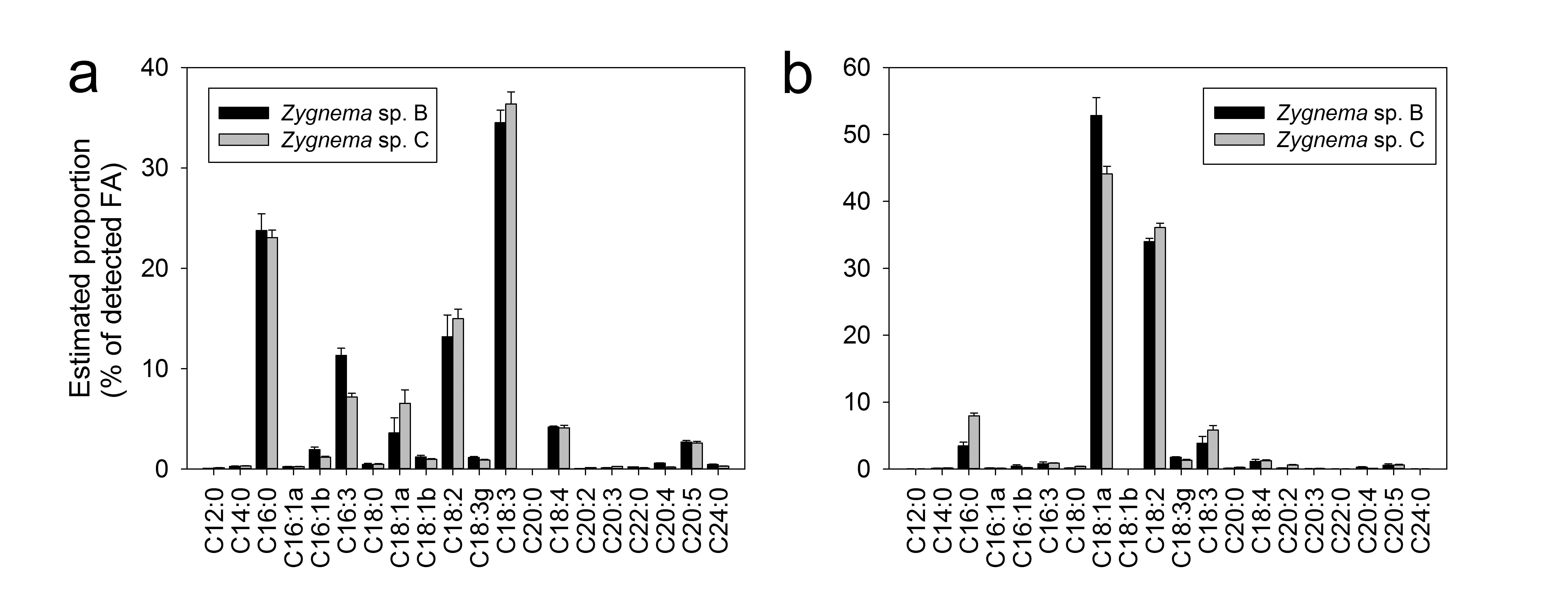

Supplement: Supplementary Data [file fiw096_supplementary_data.zip › Fig_supl_2.tif]

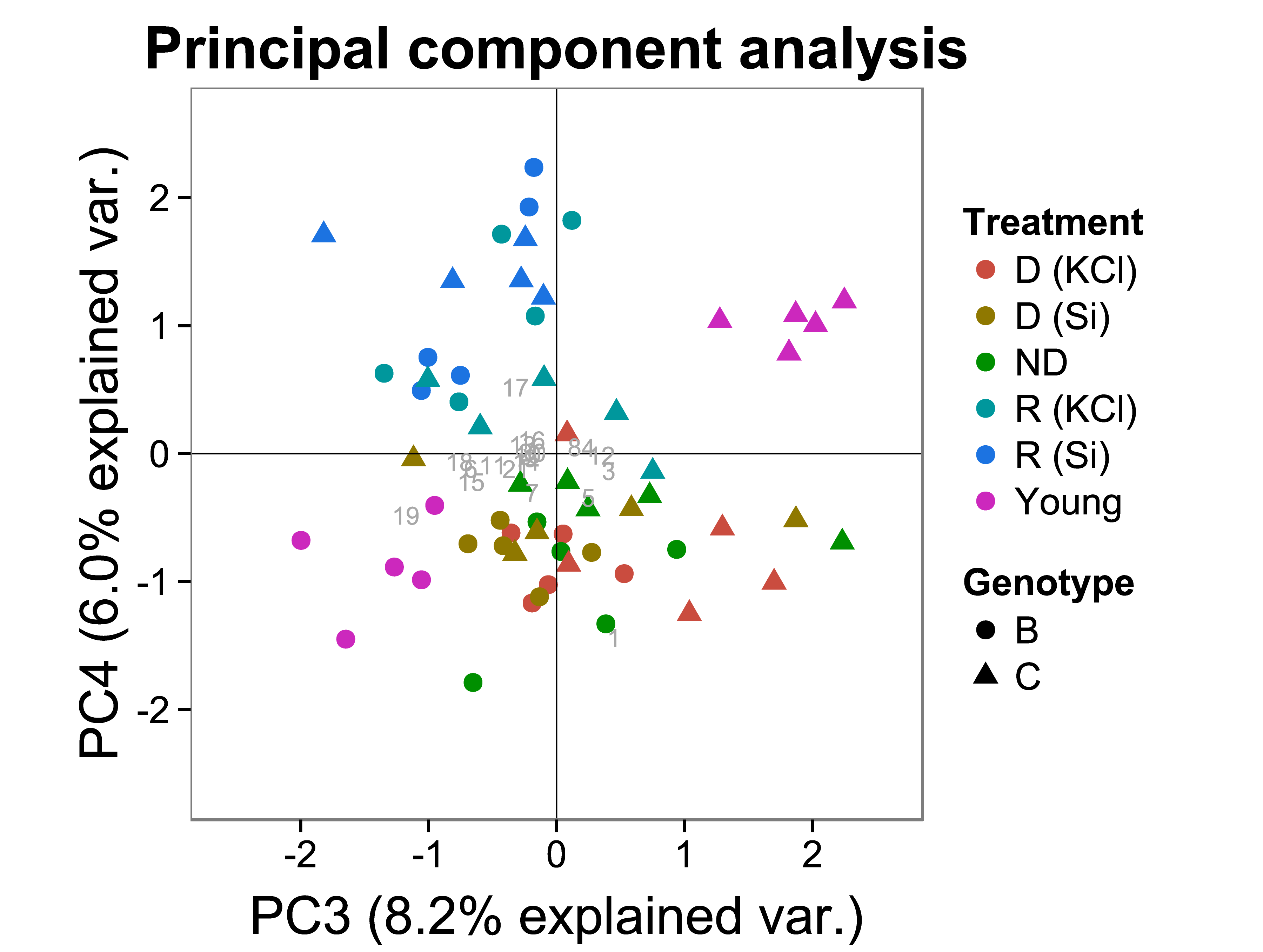

Supplement: Supplementary Data [file fiw096_supplementary_data.zip › Fig_supl_3.tif]
