# Supplementary material for: Formation of lipid bodies and changes in fatty acid composition upon pre-akinete formation in Arctic and Antarctic Zygnema (Zygnematophyceae, Streptophyta) strains
Source: FEMS Microbiol Ecol. 2016 May 10;92(7):fiw096. doi: 10.1093/femsec/fiw096 (PMC4892695; doi:10.1093/femsec/fiw096)
Supplement: Supplementary Data [file fiw096_supplementary_data.zip › Supplementary Figure Legend.doc]

**Supplementary Figure 1.** Visual assessment of living and dead cells after desiccation and rehydration treatment. (a) cells after desiccation at 86% RH, living cells marked by asterisks, (b) dead cells after desiccation at 18% RH. Scale bars 20 µm.

**Supplementary Figure 2:** Estimation of the respective proportions of the fatty acids identified in young cultures (panel a) and pre-akinetes (panel b) cultures of strains B (black bars) and C (grey bars). The proportion of each fatty acid identified was estimated based on the respective peak areas on the total ion current chromatogram. Data are means plus standard deviation (n=5).

**Supplementary Figure 3:** PCA ordination plot of samples from both strains and all experimental treatments based on their relative FA composition. The third and fourth principle components are shown. Abbreviations: D (KCl) desiccated over KCl, D (Si) desiccated over silicagel, ND not desiccated, R (KCl) desiccated over KCl and rehydrated, R (Si) desiccated over silicagel and rehydrated, Young: young cultures.
